# Family characteristics and the use of maternal health services: a population-based survey in Eastern China

**DOI:** 10.1186/s12930-016-0030-2

**Published:** 2016-10-22

**Authors:** Ling Zhang, Chengbing Xue, Youjie Wang, Liuyi Zhang, Yuan Liang

**Affiliations:** 1 Family Planning Research Institute, Tongji Medical College, Huazhong University of Science and Technology, Wuhan, China; 2 Health Statistic and Data Center of Jiangsu Province, Nanjing, China; 3 Public Health School, Tongji Medical College, Huazhong University of Science and Technology, Hangkong Road 13, Wuhan, Hubei China

**Keywords:** Maternal health care use, Pregnant women, Family characteristics, Prenatal care, Postnatal visits

## Abstract

**Background and objectives:**

Despite the benefits of maternal health services, these services are often underutilized, especially in the developing countries. The aim of the present study is to provide insight regarding factors affecting maternal health services use from the family perspective.

**Methods:**

We use data from the fourth National Health Services Survey in Jiangsu province of Eastern China to investigate the effect of family characteristics on the use of maternal health services. Family characteristics included whether or not living with parents, age of husband, husband’s education, and husband’s work status as well as family economic status. Demographic variables, social and environmental factors, and previous reproductive history were taken as potential confounders. Multiple logistic regression models were used to examine the independent effects of the family characteristic variables on maternal health service utilization.

**Results:**

The data indicate that the percentages of prenatal care, postnatal visits and hospital delivery were 85.44, 65.12 and 99.59 % respectively. Living with parents was associated with less use of prenatal care and husband’s age, education and employment status had no effect on the use of prenatal care after adjusting for potential confounding variables.

**Conclusions:**

Our findings suggest that maternal health education (especially the role of prenatal care) needs to be extended beyond the expectant mothers themselves to their parents and husbands. The difference of health care delivery as a result of traditional family culture may highlight the differences in factors influencing the use of postnatal visits and those influencing the use of prenatal care; which may be worthy of further study.

## Background

Maternal health services are important to ensure women and children’s health, which is the base of human sustainable development. Despite the benefits of maternal health services, these services are often underutilized, especially in the developing countries [1–3]. Previous research on the factors that influence the use of maternal health services has mainly focused on individual factors, such as economic status, education level, ethnicity and race, religion, attitude and knowledge about health, physical activity, and health insurance [4–9]. However, the use of health services is complex, and influenced by many factors [10, 11]. The bio-psycho-social medical model suggested that the factors affecting health need to be extended beyond the individual to society. Accordingly, this model must also be extended to the factors influencing the use of maternal health services. Furthermore, maternal health services are not only the responsibility of the pregnant woman and her doctor, but also the responsibility of the family and the whole society. Therefore, research on the use of maternal health services should extend beyond individual-level factors related to pregnant women, to include social-level factors [12].

As the basic social unit, the family has an important effect on health and health-related issues, including the use of health services [13, 14]. Although many studies have examined the importance of family-related factors on health and the use of health services, those studies mainly targeted children and adolescents [15–17]. A limited number of studies have investigated the role of the husband in the use of maternal health services, with most of these from the Western countries [18–21]. However, it is important to note that there are substantial cultural differences in family characteristics between Eastern and Western countries. These differences may influence research results about family factors related to maternal health care. In Western societies, the typical nuclear family structure comprises a husband, wife, and their non-adult children. Children aged 18 and older often live independently from their parents [22–24]. Family systems in Western societies can be redefined as married, non-married partners, separated, divorced, and remarried [25, 26]. In an Eastern society such as China, adult children and their parents often live together, including adult children who are married. This tradition (adult children living with aging parents) is termed “filial piety” in Chinese culture, and represents a core value of traditional Chinese society [23, 27, 28]. Typically in Chinese families, three or four generations (newly married young people, their parents, children born after marriage, and even grandparents of the newly married young people) live under the same roof. This family structure serves many purposes, including caring for older adults, older family members sharing housework and caring for the children of the young couple, and emotional interactions between younger and older family members. The reliance on family values based on traditional Chinese culture plays a much greater role than religious beliefs and activities, which is very different from Western societies [27–29]. These differences should not be ignored in studies of the use of maternal health care in China.

Two aspects of Chinese family characteristics of pregnant women are worth highlighting [30, 31]. First, there are few unmarried pregnant women in China, with almost all pregnant women being married with a husband. This is because being unmarried but preparing to give birth is not acceptable in Chinese society according to traditional ethics. Second, expecting the birth of a new life is a happy occasion in China. Older adults are pleased because they will soon “go up a grade” as grandparents, perhaps even becoming great-grandparents. It is generally accepted that prospective grandparents (older adults) live with their adult children who are expecting a child, providing the pregnant woman (daughter-in-law or daughter) with daily life care, and sharing their experiences of caring for a baby. However, to our knowledge, there are almost no population-based studies about the effect of family characteristics on the use of maternal health care in China.

In addition, two aspects of maternal health care in China should be noted. First, China’s maternal health care (usually including prenatal care, delivery, and postpartum hospital visits) has become an essential public health service, benefiting from national laws on population and family planning and on maternal and infant health care [31–33]. Pre- and postnatal visits are almost free of charge, and hospital delivery charges enjoy preferential government policies (are inexpensive) in maternal and child health institutions/hospitals. In China, these services are usually public. Second, in China, prenatal care usually requires pregnant women to actively go to maternal and child health institutions/hospitals. However, postnatal care consists of at-home visits by doctors, nurses, or midwives from maternal and child health institutions/hospitals, as new mothers are traditionally expected to rest indoors for 1 full month after giving birth (in Chinese “Zuo-Yue-Zi,” meaning 1-month confinement after childbirth). To a certain extent, postnatal visits are passive services. Maternal health care is not only related to physiological/medical issues, but also to psychological/social issues, which should not be ignored.

The present study offers preliminary evidence about the influence of five family characteristics variables (living with parents, husband’s age, husband’s education, husband’s employment status and family economic status) on the use of three different types of maternal health services (prenatal care, hospital delivery, and postnatal visits). Two questions are addressed: (1) What are the family characteristics of pregnant women in China? (2) What is the relationship between family characteristics and the use of maternal health care?

## Methods

### Data

**Fig. 1:**
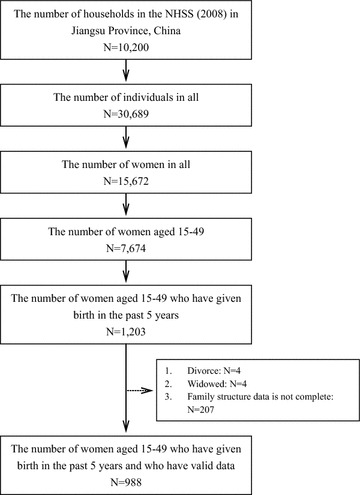
Number of respondents in each stage of study sample selection

Data used in this analysis were drawn from the Household Health Survey of the fourth China National Health Services Survey (NHSS) in Jiangsu province, Eastern China, from June 2008 to July 2008. The NHSS is organized and directed by the Center of Health Statistics and Information under the Ministry of Health, China, and has been conducted every 5 years since 1993 [34, 35]. For the fourth NHSS, the Ministry used a multi-stage, stratified cluster sampling method with systematic random sampling at each stage. The fourth NHSS had four parts: the Household Health Survey, Health Institution Survey, Prescription Survey, and Medical Staff Survey, with 1 % of the population included in each sampled province. Quality control was implemented by supervisors charged with guiding and inspecting each survey step [36, 37]. For the present study, there were 10,200 families represented in the sample, with the study population being women aged 15–49 years who had given birth since 2003. The number of respondents in each stage of the study sample selection is displayed in Fig. 1.

Jiangsu province is centrally located on the eastern coast of mainland China. The city of Shanghai and Zhejiang and Jiangsu provinces constitute the Yangtze River Delta city group. The eastern coast of mainland China is one of the most developed economic regions, and can provide a social development and health service provision model for poorer middle and west regions. The 2010 national census reported the resident population of Jiangsu province was 7865.99 million, accounting for 5.87 % of the total population, with Jiangsu province ranked the fifth most populated in China [38]. According to the China Development and Life Index published by the China Statistical Society, in 2012, Jiangsu province was ranked fourth of the 31 provinces in mainland China with a Development and Life Index of 77.02 %. [39]. Therefore, to some degree, Jiangsu province can be considered as representative of developed coastal areas in Eastern China.

### Measures

#### Dependent variables

Three variables related to the use of preventive health care by pregnant women during their last pregnancy were included in the dataset: prenatal care, hospital delivery, and postnatal visits. Inquiries pertaining to three variables began with the questions: How many times did you receive prenatal care? Did you give birth in a hospital? How many times did you receive postnatal visits?

#### Independent variables

We included five independent variables: whether or not the woman are living with parents, husband’s age, husband’s education, husband’s employment status, and family economic status. Family economic status was measured with the per capita net income (family net income/family size). In addition, given the differences between urban and rural China, the classification of economic conditions of urban and rural residents were separated [36]. Urban areas with a cash receipts income and rural areas with a net income level of P25 and below were defined as bad; P25–75 as average; and P75 and above as good.

#### Control variables

We included variables that could influence the use of maternal health care as controls to statistically eliminate their effects on the dependent variables. Pregnant women’s demographics, social and environmental factors, and previous reproductive history (which are correlated with the use of maternal health care) were regarded as control variables. Demographic variables included the pregnant women’s age, education, and employment status of the pregnant women. Previous reproductive history includes responses to the questions: How many times have you been pregnant (including abortions)? How many times have you given birth? Inquiries about social and environmental factors include the questions: Do you have health insurance? How long does it take to get to the nearest medical institution using the fastest way available? In addition, we add the types of place of residence, namely rural and urban with reference to existing studies [11, 12].

### Statistical analysis

We used three multiple logistic regression models to examine the independent effect of the family characteristic variables on maternal health service utilization: (1) the effect of the family characteristic variables shown alone, (2) with demographic, and previous reproductive history variables added, and (3) with social and environmental variables included. In addition, although some studies considered only one visit as use of prenatal care, four visits are recommended [40, 41]. The present study uses a binary dependent variable (1 = four visits or more; 0 = three visits or less). Similarly, we use a binary variable for postnatal visits (1 = one visit or more; 0 = zero visits), based on previous studies. As 99.59 % of women had given birth in the hospital, we did not perform multiple logistic analysis of hospital delivery. The coefficients from all regression models are reported as odds ratios (OR) with 95 % confidence intervals (CI). All analyses are performed using SPSS, version 12.0 (SPSS Inc, Chicago, IL, USA).

## Results

### Descriptive statistics for the primary variables

The descriptive statistics for the primary variables are presented in Table 1. Of the three variables related to preventive health care use by pregnant women, the percentage of hospital delivery is the highest (99.59 %), and that of postnatal visits is the lowest (65.12 %). The majority of pregnant women lived with their parent (68.32 %), reflecting China’s traditional family culture. In addition, it is interesting to note that family economic status did not significantly differ between pregnant women who lived with their parents and those who did not (*χ* ^2^ = 0.54; *p* = 0.46).

**Table 1:** Descriptive statistics for the primary variables (n = 988)

Variables	N	%
Times of prenatal care		
≥4	844	85.44
3 and less	144	14.56
Hospital delivery (missing = 4)		
Yes	980	99.59
No	4	0.41
Times of postnatal visits (missing = 16)		
≥1	633	65.12
0	339	34.88
Whether or not living with parents		
Yes	675	68.32
No	313	31.68
Husband’s age		
≥35 year-old	305	30.87
≥30 year-old	426	43.12
18–29 year-old	257	26.02
Husband’s education (missing = 1)		
College and above	244	24.72
High school	301	30.50
Junior high school	393	39.82
Primary school and below	49	4.96
Husband’s employment status (missing = 2)		
Yes	940	95.33
No	46	4.67
Pregnant women’s age		
≥35 year-old	193	19.53
≥30 year-old	373	37.75
≥25 year-old	370	37.45
18–24 year-old	52	5.26
Pregnant women’s education (missing = 1)		
College and above	205	20.77
High school	261	26.44
Junior high school	406	41.13
Primary school and below	115	11.65
Pregnant women’s employment status (missing = 2)		
Yes	862	87.42
No	124	12.58
Times of previous pregnancies of pregnant women		
2 and more	163	16.50
1	304	30.77
0	521	52.73
Times of previous giving births of pregnant women		
1 and more	199	20.16
0	788	79.84
Health insurance (missing = 6)		
Yes	858	87.37
No	124	12.63
The time to the nearest medical institution by the fastest way available (missing = 2)		
≥10 min or more	490	49.70
<10 min	496	50.30
Per capita income of family		
Good	311	31.48
General	433	43.83
Bad	244	24.70
Place of residence		
Rural	577	58.40
Urban	411	41.60

### Multivariate analysis of the association between family characteristics and prenatal care

Table 2 displays the ORs for the associations between independent variables and prenatal care use. In model 1, living with parents, husband’s education, and family economic status are significantly associated with prenatal care use. Specifically, respondents living with parents are less likely to report prenatal care use compared with those not living with parents (OR 0.45, 95 % CI 0.30–0.67). Model 2 shows that living with parents was the only variable that remained significant (OR 0.56, 95 % CI 0.36–0.87) with the addition of the women’s demographics and previous reproductive history. Finally, living with parents and family economic status remained significant with the addition of social and environmental variables in model 3 (OR 0.48, 95 % CI 0.30–0.77; OR 2.20, 95 % CI 1.11–4.35, respectively).

**Table 2:** Estimated net effect of family characteristic variables and control variables on the use of prenatal care and postnatal visits (n = 988)

Variables	Prenatal care			Postnatal visits		
	Model 1	Model 2	Model 3	Model 1	Model 2	Model 3
Family characteristic variables						
Whether or not living with parents (ref = No)						
Yes	0.45 (0.30–0.67)**	0.56 (0.36–0.87)**	0.48 (0.30–0.77)**	1.12 (0.82–1.53)	1.06 (0.76–1.48)	0.97 (0.69–1.37)
Husband’s age (ref = 18–29 year-old)						
≥35 year-old	0.90 (0.55–1.48)	1.40 (0.64–3.04)	1.21 (0.55–2.66)	0.84 (0.57–1.23)	0.60 (0.35–1.03)	0.53 (0.31–0.92)*
≥30 year-old	1.36 (0.83–2.22)	1.56 (0.86–2.82)	1.49 (0.82–2.70)	0.72 (0.51–1.01)	0.63 (0.42–0.95)*	0.59 (0.39–0.90)*
Husband’s education (ref = primary school and below)						
College and above	10.19 (4.26–24.34)**	2.93 (0.92–9.33)	2.17 (0.65–7.23)	2.55 (1.33–4.89)**	2.52 (1.10–5.78)*	2.04 (0.87–4.77)
High school	3.54 (1.72–7.27)**	1.53 (0.66–3.55)	1.35 (0.57–3.18)	1.78 (0.95–3.35)	2.10 (1.00–4.40)*	1.80 (0.85–3.80)
Junior high school	1.97 (1.02–3.78)*	1.56 (0.75–3.23)	1.49 (0.71–3.12)	1.48 (0.81–2.73)	1.90 (0.96–3.76)	1.76 (0.89–3.49)
Husband’s employment status (ref = No)						
Yes	1.53 (0.57–4.11)	1.02 (0.35–3.00)	0.81 (0.27–2.43)	0.71 (0.38–1.34)	0.70 (0.35–1.38)	0.63 (0.31–1.28)
Per capita income of family (ref = Bad)						
Good	2.19 (1.15–4.15)*	1.85 (0.95–3.59)	2.20 (1.11–4.35)*	0.92 (0.61–1.37)	0.90 (0.59–1.37)	1.09 (0.70–1.68)
General	1.02 (0.67–1.55)	0.90 (0.58–1.40)	0.98 (0.62–1.53)	0.95 (0.68–1.32)	0.95 (0.68–1.34)	1.03 (0.73–1.46)
Control variables						
Pregnant women’s age (ref = 18–24 year-old)						
≥35 year-old		1.01 (0.32–3.18)	1.00 (0.31–3.17)		3.60 (1.51–8.59)**	3.52 (1.46–8.50)**
≥30 year-old		0.90 (0.34–2.37)	0.85 (0.32–2.24)		1.88 (0.91–3.89)	1.78 (0.85–3.73)
≥25 year-old		1.10 (0.47–2.57)	1.08 (0.46–2.52)		1.70 (0.89–3.25)	1.72 (0.89–3.32)
Pregnant women’s education (ref = primary school and below)						
College and above		2.90 (0.99–8.56)	1.74 (0.54–5.58)		0.72 (0.35–1.50)	0.44 (0.20–0.97)*
High school		3.22 (1.46–7.09)**	2.42 (1.05–5.56)*		0.54 (0.29–0.99)*	0.40 (0.21–0.75)**
Junior high school		1.37 (0.78–2.40)	1.17 (0.66–2.08)		0.54 (0.32–0.91)*	0.46 (0.27–0.79)**
Pregnant women’s employment status (ref = No)						
Yes		1.70 (0.85–3.40)	1.70 (0.80–3.58)		1.09 (0.69–1.71)	1.01 (0.63–1.64)
Times of previous pregnancies of pregnant women (ref = 0)						
2 and more		1.27 (0.66–2.46)	1.24 (0.64–2.42)		0.58 (0.37–0.89)*	0.54 (0.35–0.84)**
1		1.66 (0.93–2.96)	1.64 (0.92–2.94)		0.68 (0.48–0.95)*	0.66 (0.47–0.93)*
Times of previous giving births of pregnant women (ref = 0)						
1 and more		2.87 (1.45–5.66)**	2.54 (1.26–5.12)**		1.44 (0.89–2.35)	1.27 (0.77–2.09)
Health insurance (ref = No)						
Yes			0.67 (0.37–1.23)			0.86 (0.56–1.33)
The time to the nearest medical institution by the fastest way available (ref = <10 min)						
≥10 min or more			1.00 (0.68–1.48)			1.26 (0.95–1.67)
Place of residence (ref = Urban)						
Rural			0.43 (0.23–0.82)*			0.50 (0.33–0.75)**

* *P* < 0.05

** *P* < 0.01 (two-tailed test)

### Multivariate analysis of the association between family characteristics and postnatal visits

Postnatal visits were associated with the family characteristic variables (Table 2), but there are some differences compared with prenatal care use. Model 1 shows that only the husband’s education was related to the use of postnatal visits. Furthermore, women whose husbands had a higher education level, specifically college and above, were more likely to report postnatal visits compared to those whose husbands had a primary school education or lower (OR 2.55, 95 % CI 1.33–4.89). With the addition of demographic factors and previous reproductive history, model 2 showed that husband’s age and education have significant effects on postnatal visits. Finally, only husband’s age remained significant, showing a negative effect with the addition of social and environmental variables in model 3 (OR 0.53, 95 % CI 0.31–0.92).

## Discussion

### Family characteristics and use of prenatal care

The results of this study show that couples living with parents accounted for the majority of respondents, which is consistent with China’s traditional culture of filial piety [27, 28]. However, it is noteworthy that living with parents was associated with less use of prenatal care. This may be explained by two reasons. First, pregnant women who live with parents may receive substantial help during daily activities, such as grocery shopping, cooking, washing clothes, and other housework, compared with those who do not live with parents. Therefore, they are likely to have less stress and fatigue related to housework. Moreover, a pregnant woman’s husband is typically the main wage earner, usually leaving home early and returning late. Therefore, pregnant women who live with parents may have more of a sense of security, feel less lonely, and enjoy a more comfortable life than those not living with their parents [27, 28]. Second, older parents, especially an older mother-in-law or mother, can provide their adult children with general knowledge about pregnancy, such as nutrition, fetal movement, and personal hygiene, as they have successfully experienced pregnancy. Therefore, the perceived need for and the use of prenatal care may be reduced. However, although the traditional family culture (living with parents) may provide support for the daily life of pregnant women, professional prenatal care should not be ignored; synergy of both types of support will be more conducive to maternal and fetal health. In addition, maternal health education (especially the role of prenatal care) needs to be extended from pregnant women to their parents.

This study shows that household income and the education of pregnant women had a positive effect on maternal health, which confirm the findings of previous studies [1–3, 12, 29]. It is noteworthy that the husband’s age and employment status were not significantly associated with the use of prenatal services. Pregnancy and the fetus are not only maternal responsibilities, but are also a husband’s responsibility. The non-significant effects we found might indicate that husbands lack awareness of their responsibilities. In addition, although age is generally a risk factor for individual disease and the use of health services, this effect may be mainly confined to the individual, and cannot be extended to others. Another possible explanation for the lack of significance of employment status may be the conflict between time and income. For example, those with jobs may have more revenue, but less time to spend with their wife; those without jobs, may have more time but less income. In other words, the conflict between time and income may confuse the impact on antenatal screening service use. The husband’s education was significantly associated with the use of prenatal services in model 1. However, it was not significantly associated with the use of prenatal services in models 2 and 3, which is partly consistent with Short and Zhang’s report that the husbands’ education level was a protective factor for prenatal care among married women in rural China [42]. A likely explanation for this protective effect is that the higher the education level, the higher the awareness of the importance of health, which affects not only the individual, but also their family, including their wife and unborn child. This suggests maternal health education also needs to be extended to pregnant women’s husbands. The policy pathway of taking husbands with higher education levels as entry points, and using these smaller populations to guide other husbands’ participation in prenatal care may be an effective and sustainable development mechanism for the use of prenatal care.

### Family characteristics and use of postnatal visits

The present study shows that living with parents, the per capita income of the family, and the husband’s employment status have no significant influence on the use of postnatal care. However, the husband’s age has a significant influence (the older the husband’s age, the lower the use of postnatal care). Factors influencing the use of postnatal visits also differed from those influencing the use of prenatal care. Living with parents, the per capita income of the family and the numbers of previous births had a significant influence on prenatal care, but not on postnatal visits. In contrast, the numbers of previous pregnancies had a significant influence on postnatal visits but not on prenatal care. These differences may be attributable to the difference of service mode between prenatal care and postnatal visits. As mentioned, in China, prenatal care is general active, whereas post-natal visit are passive. Therefore, factors affecting the use of postnatal visits may differ from those influencing the use of prenatal care. It may be inappropriate to analyze the factors influencing the use of postnatal visits with the same model of those of prenatal care, which are rarely mentioned in existing studies.

This study had several limitations. First, there was a high risk of correlation between the variables we tested (e.g., economic level and employment, or education), which might have affected the results of the study. Second, our survey was cross-sectional, and no conclusions can be drawn about causation. Furthermore, we only analyzed data for the fourth NHSS (2008) as data for other times was inaccessible (e.g., the fifth NHSS conducted in 2013). However, the present study provides a comparative basis for subsequent future analysis.

In conclusion, this study provided empirical data about family factors and maternal health services use in China. In particular, pregnant women living with parents were less likely to report prenatal care use compared with those not living with parents. The husband’s age and employment status had no effect on the use of prenatal care, after adjusting for potential confounding variables. Our findings suggest that maternal health education (especially the role of prenatal care) needs to be extended beyond the expectant mothers themselves to their parents and husbands. The difference of health care delivery as a result of traditional family culture may highlight the differences in factors influencing the use of postnatal visits and those influencing the use of prenatal care; which may be worthy of further study.
